# Geigerin-induced cytotoxicity in a murine myoblast cell line (C2C12)

**DOI:** 10.4102/ojvr.v84i1.1465

**Published:** 2017-10-31

**Authors:** Christo J. Botha, Sarah J. Clift, Gezina C.H. Ferreira, Mxolisi G. Masango

**Affiliations:** 1 Department of Paraclinical Sciences, University of Pretoria, South Africa; 2 Food, Feed and Veterinary Public Health, Agricultural Research Council-Onderstepoort Veterinary Institute, South Africa

## Abstract

*Geigeria* poisoning in sheep, locally known as ‘vermeersiekte’, is an economically important plant poisoning in southern Africa. The toxic principles contained by the toxic plants are believed to be several sesquiterpene lactones, such as geigerin, vermeeric acid and vermeerin, which cause striated muscle lesions in small stock. Because of ethical issues surrounding the use of live animals in toxicity studies, there is currently a dire need to establish an *in vitro* model that can be used to replace traditional animal experimentation. The objective of this study was to determine the cytotoxicity of geigerin in a murine myoblast cell line (C2C12) using methyl-thiazol-tetrazolium (MTT) and lactate dehydrogenase (LDH) assays, annexin V and propidium iodide (PI) flow cytometry and transmission electron microscopy (TEM). Mouse myoblasts were exposed to 2.0 mM, 2.5 mM and 5.0 mM geigerin for 24, 48 and 72 h. A concentration-dependent cytotoxic response was observed. Apoptosis was detected by means of annexin V flow cytometry during the first 24 h and apoptotic bodies were also visible on TEM. According to the LDH and PI flow cytometry results, myoblast cell membranes were not injured. We concluded that the murine myoblast cell line (C2C12) is a suitable model for future studies planned to evaluate the cytotoxicity of other and combinations of sesquiterpene lactones, with and without metabolic activation, implicated in ‘vermeersiekte’ and to elucidate the subcellular effects of these myotoxins on cultured myoblasts.

## Introduction

*Geigeria* poisoning, locally referred to as ‘vermeersiekte’, is an important plant poisoning in southern Africa and in certain seasons, large numbers of sheep have been affected (Grosskopf 1964; Kellerman, Naudé & Fourie 1996). All plants of the genus *Geigeria* may cause intoxication, but there are two species which are of particular economic importance in southern Africa, namely, *Geigeria ornativa* and *Geigeria aspera* (Kellerman et al. 2005). The toxicity induced by these *Geigeria* species is attributed to various sesquiterpene lactones and several have been isolated; for example, vermeeric acid (Rimington, Roets & Steyn, 1936), geigerin (Rimington & Roets 1936), geigerinin (De Villiers 1959) and vermeerin (Grosskopf 1964). Rimington et al. (1936) proposed that vermeeric acid is the cause of vermeersiekte, but inexplicably the compound could not be re-isolated by various workers (Grosskopf 1964; Kellerman et al. 2005). In a preliminary trial, Rimington and Roets (1936) drenched geigerin to only one sheep, without ill effect; however, subcutaneous injection and oral dosing to cats induced toxicity. Thus, it is generally believed that a combination of different sesquiterpene lactones induces poisoning (Kellerman et al. 2005). The oral administration of an extract containing geigerin, ivalin and dihydrogriesenin to sheep induced ‘vermeersiekte’ (Kellerman et al. 2005). The plant toxins are cumulative, as under natural conditions sheep have to graze *Geigeria ornativa* for approximately 2–3 weeks before clinical signs are noticed (Grosskopf 1964; Kellerman et al. 1996). Furthermore, in experimental studies to reproduce the syndrome, sheep received plant material over lengthy periods before exhibiting clinical signs (Botha et al. 1997; Pienaar et al. 1973; Van Heerden, Van der Lugt & Durante 1993). However, progress to elucidate the specific or combination of toxic sesquiterpene lactones that will induce ‘vermeersiekte’ has been hampered because of the lack of a suitable small laboratory animal model. Such a model would be required to test the isolated fractions because it would simply not be feasible to prepare sufficient quantities of the fractions for administration to sheep over extended periods (Grosskopf 1964).

The overarching aim was to establish an *in vitro* tissue culture model to replace the use of sentient animals in toxicity testing, which can then be used in future studies to screen other and/or combinations of sesquiterpene lactones implicated in ‘vermeersiekte’ for cellular toxicity. The specific objective of this study was to investigate the cytotoxicity induced by geigerin in a mouse myoblast cell line (C2C12). Cytotoxicity of geigerin was determined using the methyl-thiazol-tetrazolium (MTT) and lactate dehydrogenase (LDH) assays as well as annexin V and propidium iodide (PI) flow cytometry. Transmission electron microscopy (TEM) was used to investigate subcellular changes induced by geigerin *in vitro*.

## Materials and methods

### Chemicals and reagents

All the chemicals, reagents and cell culture media were purchased from Sigma Aldrich (South Africa) unless otherwise stated. Cell culture flasks and plates were purchased from AEC Amersham (South Africa).

### Geigerin

**FIGURE 1:**
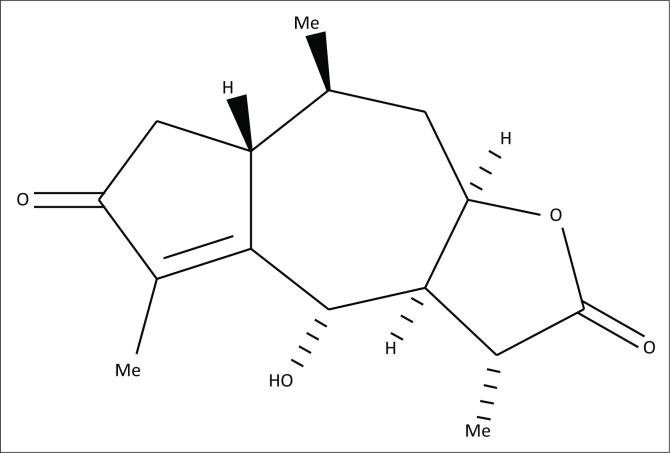
Geigerin, a sesquiterpene lactone, isolated from *Geigeria aspera*.

Geigerin, isolated from *G. aspera* in the 1980s, has been preserved in dried form in a locked safe deposit as part of the natural toxin collection of the Department of Paraclinical Sciences, Faculty of Veterinary Science, Onderstepoort. Before commencement of the study, a sub-sample of the pure compound was sent to the Department of Chemistry, University of Pretoria, for structural verification by nuclear magnetic resonance (NMR). It was confirmed that the geigerin had not degraded (Figure 1). Geigerin stock solutions were prepared in acetone. The final acetone concentration did not exceed 0.5%. The working solution was prepared using the corresponding cell culture medium.

### Cell cultures

The murine skeletal muscle C2C12 cell line (CRL-1772) was obtained from the American Type Culture Collection (ATCC, Manassas, VA). The myoblasts were grown in Dulbecco’s Modified Eagle’s Medium (DMEM), supplemented with 10% foetal calf serum (FCS) and 4 mM glutamine, in a humidified atmosphere of 5% CO_2_ at 37 °C. The cells were cultured in 75 cm^2^ cell culture flasks.

### Cytotoxicity assays

#### Exposure of C2C12 cell cultures to geigerin

The cell cultures were seeded at a density of 1 × 10^6^ cells/well in 6-well microtitre plates for flow cytometry and at 2 × 10^3^ cells/well in 96-well plates for the MTT and lactate dehydrogenase (LDH) assays. After 24 h of seeding the cells, geigerin at concentrations of 2.0 mM, 2.5 mM and 5.0 mM were added to each experimental well. The plates were then incubated for 24, 48 and 72 h in a humidified atmosphere of 5% CO_2_ at 37 °C. Control wells contained cells and the corresponding culture medium only. Cells exposed to Triton-X (0.01%) and staurosporine (1 µM) were used as internal quality controls (positive controls) for necrosis (LDH and PI flow cytometry) and apoptosis (annexin V flow cytometry) assays, respectively.

#### Methyl-thiazol-tetrazolium assay

The 3-(4,5-dimethylthiazol-2-yl)-2,5-diphenyl-2*H*-tetrazolium bromide (MTT) assay was based on the modified method described by Mosmann (1983). At the end of the exposure period, the supernatants in each well were discarded and the wells were washed with 200 µL phosphate buffered saline (PBS). Then DMEM (200 µL), containing 5% FCS and 30 μL of MTT (5 mg/mL in PBS), was added to each well. The plates were gently shaken and incubated for 2 h at 37 °C in 5% CO_2_ atmosphere. The supernatants were discarded and 100 μL dimethyl sulphoxide (DMSO) was added and the plates were gently shaken for 5 min to solubilise the formed formazan. Absorbance (570 nm) was measured using an enzyme-linked immunosorbent assay (ELISA) reader (Synergy HT, BIO-TEK Instruments, Winooski, VT, USA). Cell survival was calculated as a percentage of the geigerin tested relative to the negative controls (cells exposed to media only). Three independent experiments were carried out with three replicate wells for each geigerin concentration.

#### Lactate dehydrogenase assay

Damaged plasma membranes, indicated by LDH release, were evaluated by measuring LDH activity using the CytoTox-One homogeneous membrane integrity assay kit (Promega, Madison, WI). At the end of the exposure period, 100 µL of the cell culture medium was removed from each experimental well and transferred into a 96-well opaque-walled tissue culture plate. An equal amount (100 µL) of the CytoTox-One reagent was added to the wells and the plate was incubated overnight at room temperature. At the end of the incubation, 50 µL of the stop solution was added to each well and absorbance was measured using an ELISA reader (Synergy HT, BIO-TEK Instruments, Winooski, VT, USA) at 540 nm excitation and 590 nm emission wavelengths. LDH release was calculated as the percentage absorbance of each sample relative to the negative controls (cells exposed to media only). Two independent experiments were carried out with two replicate wells for each geigerin concentration.

#### Annexin V flow cytometry assay

An annexin V-fluorescein isothiocyanate (FITC) apoptosis detection kit (Life Technologies, South Africa) was used to detect apoptotic cells. At the end of the exposure period, cells were trypsinised with trypsin-versene ethylenediaminetetra-acetic acid (EDTA), washed with Dulbecco’s PBS (DPBS) and centrifuged at 950 g for 3 min. The supernatant fluid was carefully discarded and pellets were resuspended in 1 mL DPBS and centrifuged as before. The pelleted cells were then resuspended in 300 µL of the annexin binding buffer. A cell suspension of 100 µL was transferred to sterile 2 mL Eppendorf tubes. An amount of 5 µL annexin V solution was added to each cell suspension and the tubes were incubated for 15 min at room temperature. After the incubation period, 400 µL of binding buffer was added to each tube. Samples were mixed gently and kept on ice. Fluorescence was measured immediately using a flow cytometer (Cytomics FC 500, Beckman Coulter, Miami, FL) and analysed using the Kaluza 1.1 software package. The induction of apoptosis was recorded as annexin V binding activity (%). Two experiments were carried out for each geigerin concentration.

#### Propidium iodide flow cytometry assay

PI was used to stain cells with damaged plasma membranes. At the end of the exposure period, cells were trypsinised, washed with DPBS and centrifuged as described for the annexin V assay. The supernatant was carefully discarded and pellets were resuspended in 1 mL DPBS and centrifuged as before. The pelleted cells were then resuspended in 300 µL of the binding buffer. A cell suspension of 100 µL was transferred to sterile 2 mL Eppendorf tubes. An amount of 1 µL PI solution was added to each cell suspension and the tubes were incubated for 15 min at room temperature. After the incubation period, 400 µL of binding buffer was added to each tube. Samples were mixed gently and kept on ice. Fluorescence was measured immediately using a flow cytometer (Cytomics FC 500, Beckman Coulter, USA) and analysed using the Kaluza 1.1 software package. The PI uptake was estimated as the percentage fluorescence of each sample relative to the controls. Two experiments were carried out for each geigerin concentration.

#### Transmission electron microscopy

The cell cultures were seeded at a density of 2.5 × 10^5^ cells/well in 6-well microtitre plates and after 24 h incubation, 2.0 mM, 2.5 mM and 5.0 mM geigerin were added to each experimental well. The plates were then incubated for a further 24, 48 and 72 h at 37 °C. Control wells contained cells and the corresponding culture medium only.

Subcellular changes in the myoblasts were investigated following the modified method of Glauert (1980). At the end of the exposure period, cells were fixed in 2.5% glutaraldehyde in sodium phosphate buffer for 15 min before detachment using a cell scraper. Loose cells were transferred to 2 mL Eppendorf tubes, fixed for another 1 h and pelleted using centrifugation at 830 g for 3 min. The pelleted cells were post-fixed in 1% aqueous osmium tetroxide for 1 h, followed by washing and dehydration in buffer and graded alcohols. Cells were subsequently embedded in absolute resin at 60 °C. Following overnight curing, ultra-thin sections were prepared and stained with lead citrate and uranyl acetate. The prepared sections were viewed using a transmission electron microscope (Philips CM10) operated at 80 kV.

### Statistical analysis

Basic descriptive statistics were performed (mean ± SEM) and data were expressed as percentages compared to the negative controls. Linear mixed model analysis, also known as restricted maximum likelihood (REML) analysis, was applied to the MTT activity values to model the correlation over 72 h in an analysis of repeated measurements. A combined analysis over 3 weeks was performed to test for differences between the three time periods, the three different concentrations and the period by concentration interaction effects. An antedependence model of order 1 was found to best model the correlation over time, allowing for variances to change over time. Means were compared using Tukey’s test at 5% level. All data were analysed using the statistical programme GenStat^®^ (Payne 2015).

## Results

### Methyl-thiazol-tetrazolium assay

**FIGURE 2:**
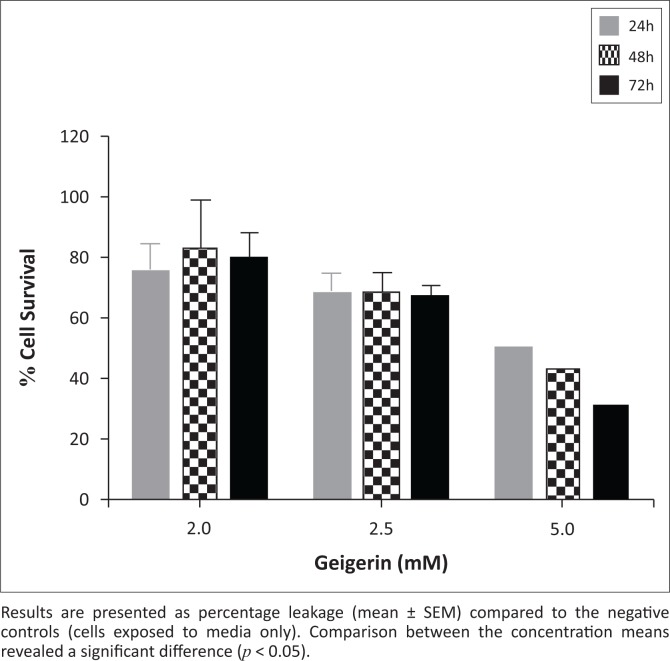
Assessment of cell survival using methyl-thiazol-tetrazolium assay following exposure of C2C12 myoblasts to geigerin (2.0 mM, 2.5 mM and 5.0 mM) for 24, 48 and 72 h.

A concentration-dependent cytotoxic response was recorded following the exposure of C2C12 myoblasts to geigerin at 2.0 mM, 2.5 mM and 5.0 mM concentrations (Figure 2). Comparison between the concentration means revealed a significant difference (*p* < 0.05), but no statistical difference occurred when the three incubation periods were compared. The greatest reduction in cell survival (%) was observed when the C2C12 cells were exposed to 5.0 mM geigerin after 24 h (49.6 ± 1.8%); 48 h (43.3 ± 1.2%) and 72 h (31.2 ± 1.2%).

### Apoptosis analysis

**FIGURE 3:**
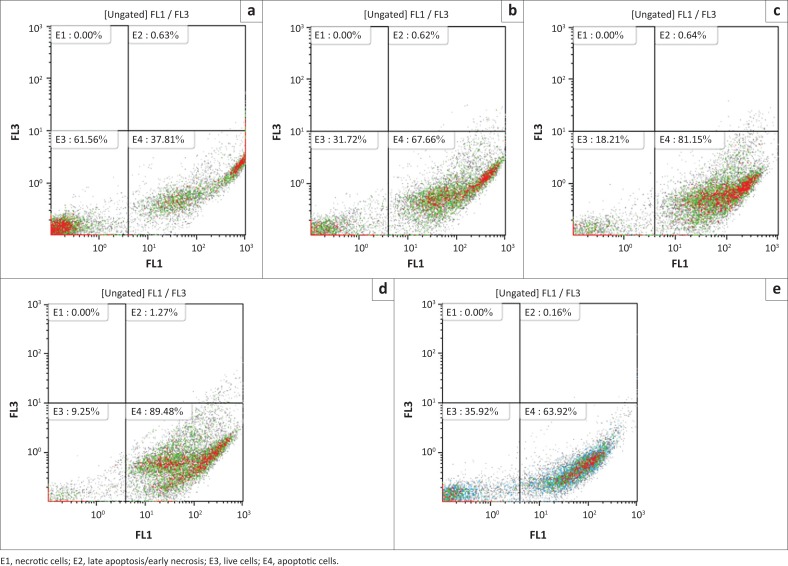
Annexin V activity (flow cytometry) following exposure of C2C12 myoblasts to geigerin (2.0 mM, 2.5 mM and 5.0 mM), media only (control) and staurosporine (positive control) for 24 h. (a) Control, (b) Geigerin 2.0 mM, (c) Geigerin 2.5 mM, (d) Geigerin 5.0 mM and (e) Straurosporine 1 µM.

Myoblasts undergoing apoptosis after exposure to geigerin for 24, 48 and 72 h were detected using the annexin V flow cytometry assay. A concentration-dependent increase in annexin V binding activity was recorded only at the 24 h exposure period (Figure 3) for the different geigerin concentrations (i.e. 67.66% for 2.0 mM; 81.15% for 2.5 mM; 89.48% for 5.0 mM) when compared to the negative controls (37.81%). These results indicate that apoptosis was induced in the myoblasts exposed to geigerin for 24 h.

### Necrosis analysis

**FIGURE 4:**
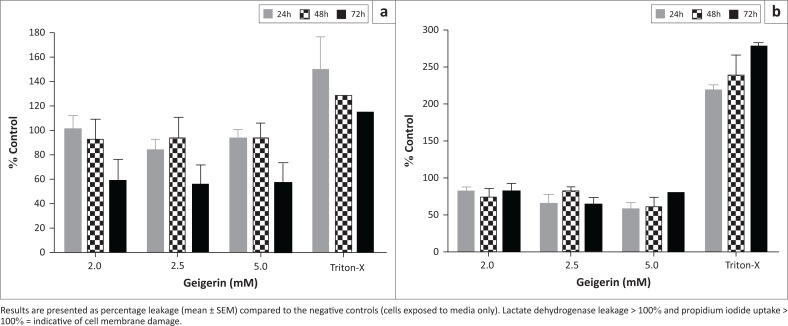
(a) Lactate dehydrogenase enzyme leakage and (b) propidium iodide uptake (flow cytometry), following exposure of C2C12 myoblasts to geigerin (2.0 mM, 2.5 mM and 5.0 mM), media only (control) and Triton-X (positive control) for 24, 48 and 72 h.

The induction of necrosis following exposure of the C2C12 myoblasts to geigerin at 2.0 mM, 2.5 mM and 5.0 mM concentrations for 24, 48 and 72 h was investigated using the LDH and PI flow cytometry assays. Insignificant LDH leakage (less than 20%) was recorded after 24 and 48 h exposure to geigerin, but no LDH leakage was measured after 72 h (Figure 4a). Uptake of the PI dye by the myoblasts was also not substantial when compared to that of the control cells (Figure 4b). Collectively, these results indicate that there was very little damage to the cell membranes of the C2C12 cells following exposure to geigerin at the different concentrations and exposure periods.

### Transmission electron microscopy

**FIGURE 5:**
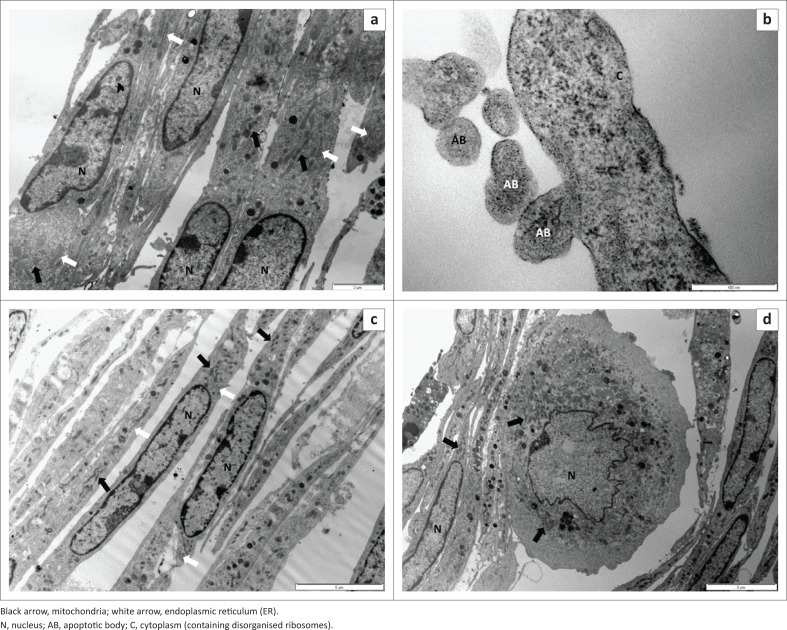
Electron micrographs showing C2C12 myoblasts exposed to 5.0 mM geigerin for 24, 48 and 72 h. (a) Control, (b) Geigerin 5.0 mM (24 h), (c) Geigerin 5.0 mM (48 h) and (d) Geigerin 5.0 mM (72 h).

Ultrastructural examination of the cultured myoblasts revealed regular apoptotic changes, namely, blebbing of the cell membrane with formation of apoptotic bodies as well as nuclear invagination and chromatin condensation towards the nuclear periphery following exposure to geigerin for 24 h when compared to the control cells (Figure 5). In general, mitochondrial membranes remained intact throughout the exposure period. The integrity of the cell and nuclear membranes remained intact throughout the exposure period similar to the control cells (Figure 5). The Golgi bodies and endoplasmic reticula (ER) appeared normal and evenly distributed in the cytoplasm of the cells. After 72 h exposure to geigerin, the ER vesicles became enlarged and the ribosomes appeared disorganised and detached when compared to control cells (Figure 5). With prolonged exposure to 5.0 mM geigerin, the mitochondrial matrix (including the cristae) became less electron-dense compared to the control cells. Rare necrotic cells (i.e. swollen or enlarged because of hydropic change, nuclear membrane indentation and loss of cell membrane contiguity) were noticed following exposure of the cells to the highest concentration of geigerin (5.0 mM) for 72 h (Figure 5).

## Discussion

The cytotoxicity results obtained using the MTT assay indicated that geigerin affected the activity of the mitochondrial dehydrogenase enzyme (Figure 2). The inhibition of mitochondrial oxidative phosphorylation by sesquiterpene lactones isolated from *G. aspera* has been demonstrated previously (Narasimhan, Kim & Safe 1989; Van Aswegen, Vermeulen & Potgieter 1979). Decreased enzyme activities (such as mitochondrial succinate dehydrogenase involved in mitochondrial electron transfer chain reactions) as a result of mitochondrial damage have been linked to reduced ATP synthesis, which is crucial for metabolic functions and growth of cells (Schulze-Osthoff et al. 1992).

Apoptosis is an important, highly-conserved and well-controlled process of cell death that is induced by a variety of physiological and pathological conditions (Zhang et al. 1998). It has been demonstrated that specific death receptors (e.g. Fas, tumour necrosis factor) and mitochondria both play a critical role in the initiation of apoptosis via their activation of caspases (cysteine-requiring proteases) (Sawai & Domae 2011). Phosphatidylserine, an amino-phospholipid located on the inner surface of the plasma membrane, has been used as an early marker of cells undergoing apoptosis (Krysko, D’Herde & Vandenabeele 2006). During apoptotic cell death, phosphatidylserine is actively translocated to the outer surface of the plasma membrane, where it can be detected by using annexin V (Masango, Ellis & Botha 2015). In the current study, apoptosis was induced in the C2C12 myoblasts only at the 24 h period following exposure to geigerin (Figure 3). Previous studies have reported induction of apoptosis by sesquiterpene lactones in cultured cells (Dirsch, Stuppner & Vollmar 2001; Zhang, Ong & Shen 2004; Zhang et al. 2005, 2013). The presence of apoptotic cells was furthermore confirmed by TEM after exposure to geigerin (Figure 5). Necrosis was not observed when utilising *in vitro* assays following exposure of C2C12 myoblasts to geigerin (Figure 4), but with TEM a few necrotic cells were observed at the highest exposure level after 72 h (Figure 5).

Moderately high geigerin concentrations were required to induce cytotoxicity in this study. It could be because of the cumulative effect of sesquiterpene lactones associated with this disease. Sheep have to ingest large quantities of the plant material over prolonged periods of time before they develop clinical signs (Botha et al. 1997; Grosskopf 1964; Kellerman et al. 1996). The established murine myoblast cell line could also be used to compare the relative toxicities of other more toxic αβ-unsaturated-γ-lactones, such as geigerinin, ivalin and vermeerin, also implicated as a cause of the disease (Grosskopf 1964; Kellerman et al. 2005; Rodriguez, Towers & Mitchell 1976). In addition, metabolic activation with S9-mix should also be done in order to establish if the biotransformed sesquiterpene lactones are more toxic (Hostanska et al. 2014).

## Conclusion

In summary, exposure of the C2C12 myoblasts to increasing geigerin concentrations resulted in concentration-dependent cytotoxicity. Apoptosis was the main mechanism through which geigerin-induced the observed cell death. Some ultrastructural lesions typical for necrosis were also observed at the highest geigerin exposure levels after 72 h. Based on the cytotoxicity observed, supported by TEM findings, it is concluded that the murine myoblast cell line (C2C12) could be used as a suitable *in vitro* model to evaluate cytotoxicity induced by other and/or combinations of sesquiterpene lactones implicated in ‘vermeersiekte’ in sheep, with and without metabolic activation. Future studies should also investigate subcellular effects of these myotoxins on the differentiated myotubes and/or other cell lines.
